# E-Cadherin: An Important Functional Molecule at Respiratory Barrier Between Defence and Dysfunction

**DOI:** 10.3389/fphys.2021.720227

**Published:** 2021-10-04

**Authors:** Hasan Yuksel, Merve Ocalan, Ozge Yilmaz

**Affiliations:** ^1^Department of Pediatric Allergy and Pulmonology, Faculty of Medicine, Celal Bayar University, Manisa, Turkey; ^2^Department of Pediatric Allergy and Immunology, Faculty of Medicine, Celal Bayar University, Manisa, Turkey

**Keywords:** E-cadherin, respiratory epithelial barrier, epithelial cells, adherens junction, epithelial mesenchymal transition

## Abstract

While breathing, many microorganisms, harmful environmental particles, allergens, and environmental pollutants enter the human airways. The human respiratory tract is lined with epithelial cells that act as a functional barrier to these harmful factors and provide homeostasis between external and internal environment. Intercellular epithelial junctional proteins play a role in the formation of the barrier. E-cadherin is a calcium-dependent adhesion molecule and one of the most important molecules involved in intercellular epithelial barier formation. E-cadherin is not only physical barrier element but also regulates cell proliferation, differentiation and the immune response to environmental noxious agents through various transcription factors. In this study, we aimed to review the role of E-cadherin in the formation of airway epithelial barier, its status as a result of exposure to various environmental triggers, and respiratory diseases associated with its dysfunction. Moreover, the situations in which its abnormal activation can be noxious would be discussed.

## What Is the Respiratory Epithelial Barrier

### Bronchial Epithelial Barrier Function

In humans, respiratory tract starts from nasal orifice and ends in the alveoli. Along this tract, many microorganisms, harmful environmental particles, allergens, and environmental pollutants are exposed by breathing. The airway is covered with epithelial cells. The conducting part of the respiratory tract is lined by pseudostratified columnar ciliated epithelium (from the nasal orifice to the bronchi). Terminal bronchi are lined with non-ciliated cuboidal cells. The alveoli are also lined by a thin layer of squamous epithelial cells. (Type 1 pneumocytes form simple squamous, Type 2 pneumocytes form cuboidal epithelium).

The bronchial pseudostratified mucociliary epithelium acts as a highly regulated physical barrier against the outside world, as well as in the skin and gastrointestinal tract. It blocks invasion of inhaled environmental toxic agents such as pathogen microorganisms, air pollutants, and aeroallergens. It is also is a chemical barrier that secretes mucus and thus does not allow the passage of inhaled foreign particles. In addition, airway epithelial cells have a critical role in the innate immune system. Epithelial cells produce chemokines, cytokines anti-microbial peptides, that activate another immune system cells, and promote pathogen phagocytosis and establish mucociliary clearance (Schleimer et al., 2007).

### Alveolar Epithelial Barrier Function

**FIGURE 1:**
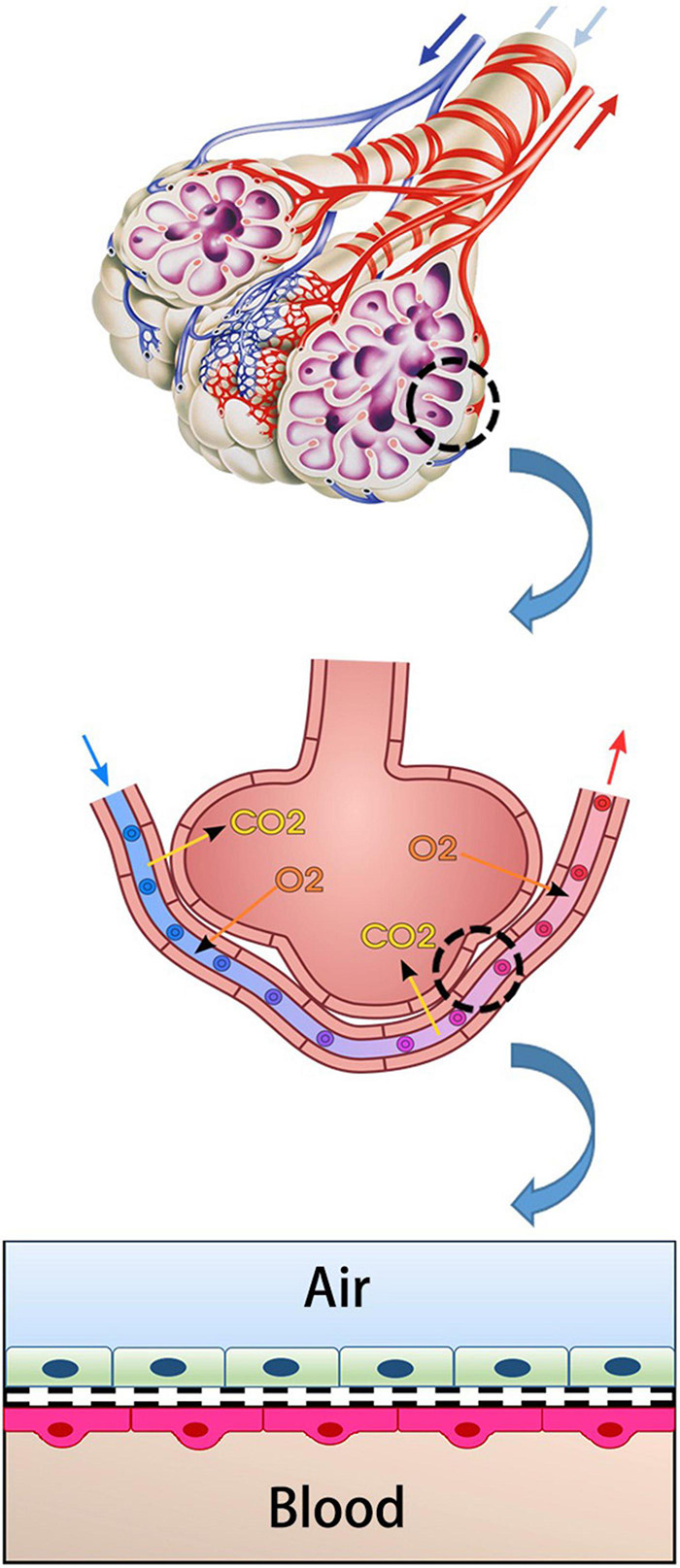
Simplified diagrammatic look of air-blood barrier.

The main unit of gas exchange is the alveoli in the human lung. The alveolo-capillary barrier provides both O_2_/CO_2_ exchange and protection against inhaled irritant particles. This structure consists of capillary endothelium and alveolar epithelium and interstitial tissue between them (Figure 1; Weibel and Knight, 1964; Hou et al., 2019). The alveolar epithelium protects the lung parenchyma from noxious agents inhaled from the external environment (Nishida et al., 2017). Lung endothelium is the side that allows the gas exchange to pass into the blood. It is an active barrier to gas exchange. Furthermore, it plays a critical role in regulating the permeability of this barrier in acute and/or chronic inflammation (Aird, 2007; Lu et al., 2018).

## Structure of Respiratory Epitelial Barrier and E-Cadherin

**FIGURE 2:**
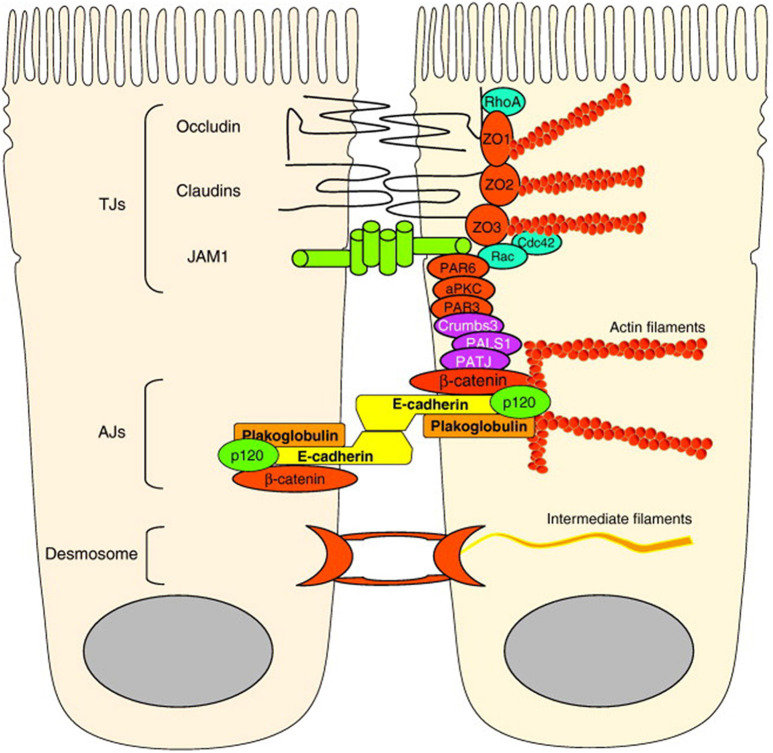
Molecular structure of airway epithelial barrier: TJs are localized apically and include transmembrane proteins claudins, occludin and JAMs. JAMs bind the cell polarity proteins Par-3 and Par-6. AJs localization is below the TJs and more basolaterally and their main component is E-cadherin, which interacts with the cytoplasmic proteins p120 catenin, β-catenin, and α-catenin to connect with the actin cytoskeleton. Desmosomes are also localized most basolaterally.

The airway epithelial barrier comprised of airway surface liquids, mucus, and intercellular epithelial junctions that form between neighboring cells (Georas and Rezaee, 2014). Intercellular epithelial junctions provide cell contact, cell polarity, and transcellular ion transport with adhesive forces (Nawijn et al., 2011; Georas and Rezaee, 2014). These intercellular junctions consist of tight junctions (TJs), adherens junctions (AJs), and desmosomes (Figure 2; Nawijn et al., 2011).

Tight junctions are considered to be the main regulators of paracellular transport of ions and solutes. Therefore, it constitutes the most important part of the physical barrier function of the epithelium (Kojima et al., 2013). TJs are localized apically. TJ proteins contain three major transmembrane proteins: (1) members of claudin family, (2) tight junction–associated MARVEL protein family members: occludin, tricellulin, and MARVELD3 (3) immunoglobulin-like proteins, such as junctional adhesion molecule (JAM) and coxsackie adenovirus receptor (CAR) (Schulzke and Fromm, 2009). JAMs bind the cell polarity proteins Par-3 and Par-6. Occludin and claudins are also connected to the cytoskeleton by zonula occludens (ZO)-1, ZO-2, and ZO-3, and cingulin (Nawijn et al., 2011).

Adherens junctions are mainly responsible for cell-cell adhesion. AJs localization is below the TJs and more basolaterally. The main component of AJs structure is E-cadherin. E-cadherin is a type I cadherin transmembrane glycoprotein and a calcium-dependent molecule. It consists of an extracellular domain that forms adhesions between epithelial cells and a cytoplasmic domain that is connected by the actin cytoskeleton. This cytoplasmic domain is stabilized at the cell membrane when connected with the anchor proteins p120 catenin, β-catenin, and α-catenin (Leckband and Prakasam, 2006; Pokutta and Weis, 2007). This binding is crucial in the formation of apical junctional complexes (AJCs) and the epithelial barrier. In addition, E-cadherin regulates cell differentiation and proliferation through various transcription factors including ZO-1-associated nucleic acid binding protein (Bajpai et al., 2008).

Desmosomes are located in the most basolateral area and distributed on the lateral surface of the epithelial cells (Godsel et al., 2004). They provide mechanical strength to tissues. Desmosomes contain non-classical cadherins and form adhesive connections with the intermediate filament of the cells (Nawijn et al., 2011).

E-cadherin is an junctional molecule that is synthesized from epithelial cells and forms the main structure of AJCs. When E-cadherin is not expressed in the epidermis, the TJ proteins such as ZO-1, occludin, and claudins are also not formed properly. In previous studies, siRNA knockdown of E-cadherin caused decreased ZO-1 expression and reduced epithelial resistance in bronchial epithelial cells (Heijink et al., 2010a).

**FIGURE 3:**
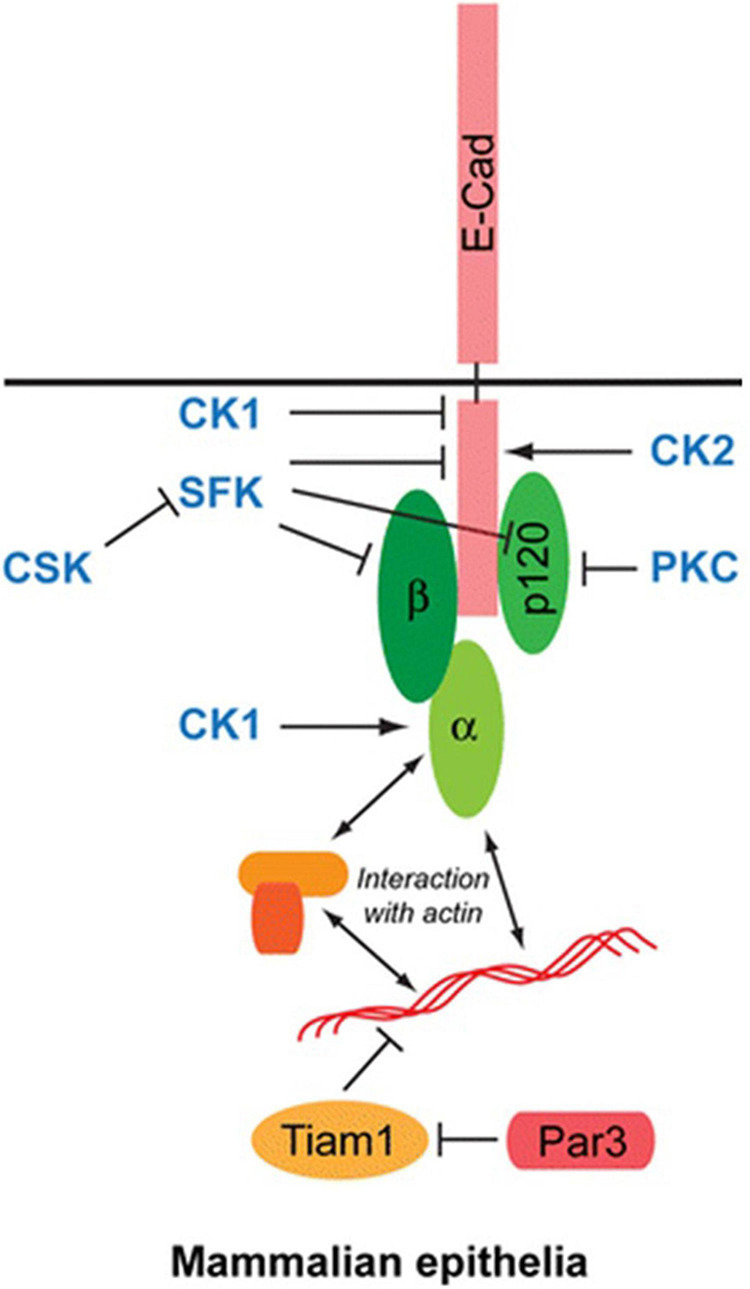
Structure of E-cadherin molecule: The cytoplasmic domain of E-cadherin interacts with two main molecules: β-catenin (β-Cat) and p120-catenin (p120-Ctn). Binding of β-Catenin with actin filaments is mediated by α-Catenin (α-Cat).

The cytoplasmic domain of E-cadherin interacts with two main molecules: β-catenin (β-Cat) and p120-catenin (p120-Ctn). The connection with the actin cytoskeleton is provided mainly by β-Cat. Binding of β-Catenin with actin filaments is also mediated by α-Catenin (α-Cat) (Figure 3; Coopman and Djiane, 2016). Many studies have shown that p120-Ctn has a crucial role in the regulation of AJs (Kourtidis et al., 2013). p120-Ctn regulates E-cadherin stability and provides cell–cell adhesion in epithelial cells. E-cadherin and catenins protein/protein interactions and their levels at the membrane are regulated by AJ-regulating kinases and phosphatases. Src kinase and other Src family kinases (SFK) are one of them. They regulate the interactions of E-cadherin and catenins. The Src kinase phosphorylates E-cadherin on two consecutive tyrosine residues in mammalian cells. Thereby it creates an interaction domain with the E3-ubiquitin ligase Hakai to mediate disruption of the E-cadherin complex (Fujita et al., 2002). SFKs also phosphorylate β-Cat and p120-Ctn (Hu et al., 2001; Kourtidis et al., 2013). As a result of phosphorylation of β-Cat, the interaction between E-cadherin and α-Cat is disrupted. Another important kinase in the regulation of E-cadherin/catenin complexes is Casein kinase 2 (CK2). E-cadherin is phosphorylated by CK2, thereby increasing its binding with β-catenin and strengthening cell-cell adhesion (Lickert et al., 2000). In the light of these data, E-cadherin is a very important regulatory protein in the formation and maintenance of epithelial cell-cell contacts.

## Effect of External Noxious Stimuli on Epithelial Barrier and E-Cadherin

The environment is an important determinant for human health. With the increase of industrialization in recent years, cigarette smoke and air pollutants such as ozone, exhaust gas has increased and air quality has decreased drastically. At the same time, increased use of detergents and cleaning products, increased consumption of food additives and emulsifiers, and the widespread use of microplastics and nanoparticles, increased the environmental toxic burden. In some of epidemiologic previous studies, an association has been found between exposure to cleaning products and increased risk of asthma (Siracusa et al., 2013). In addition, recent reports have identified that adverse environmental conditions increase respiratory tract diseases and sensitivity to some allergens (Eguiluz-Gracia et al., 2020; Itazawa et al., 2020). The effect of environmental factors on the epithelial barrier structure is summarized in Table 1.

**TABLE 1:** The effect of environmental factors on the epithelial barrier structure.

**Environmental factors**		**Effects on epithelial barrier structure**	**References**
Allergens	House-dust mite (HDM)	Induction proteolysis of ZO-1, occludin and other TJs proteins Disruption of E-cadherin and AJ structure by activating PAR 1 and/or 2	Wan et al., 2001; Matsumura, 2012; Li et al., 2019
	Pollens	Disruption E-cadherin and TJ proteins such as occludin, claudin-1 Poa pratensis, Betula, Ambrosia decreased expression of TJ proteins Olive, cypress (Italian cypress), orchard grass (Dactylis glomerata) affected both epithelial junction function and structure Phleum pratense did not affect TEER or expression of ZO-1 in human bronchial epithelial cells	Runswick et al., 2007; Vinhas et al., 2011; Blume et al., 2013; Hosoki et al., 2015
	Aspergillus fumigatus	Disruption epithelial cell detachment by production of IL-6, IL-8, and monocyte chemoattractant protein 1 from airway epithelial cells	Chaudhary and Marr, 2011
	Cockroach	Disruption of epithelial barrier by increasing IL-8 expression and activation PAR-2	Page et al., 2003
Detergents	Anionic surfactants	Disruption the structure of TJs by cleaving occludin and ZO-1	Medina-Ramon et al., 2005; Wang et al., 2019
	Laundry detergents	Increasing in IL-33 and TSLP in bronchial epithelial cells	Wang et al., 2019
Cigarette smoke		Downregulation of gene expression of E-cadherin, occludin, some claudins (such as claudin-1, 3, 4, 7, 15), and ZO-1 Dysfunction epithelial barrier and epithelial mesenchymal transition (repeated exposure)	Zou et al., 2013; Aghapour et al., 2018; Hou et al., 2019; Tatsuta et al., 2019
Ozone	Acute exposure	Epithelial barrier dysfunction, airway inflammation, peribronchial collagen deposition, and airway hyperresponsiveness	Michaudel et al., 2018b; Sokolowska et al., 2019
	Chronic exposure	Remodeling, fibrosis, and emphysema in the small airway	Michaudel et al., 2018a
PMs		Damaging structure of occludin, claudin-1, and ZO-1 downregulating claudin-1 expression in airway epithelial cells Suppression of E-Cadherin	Caraballo et al., 2011; Chuang et al., 2015; Liu et al., 2019

Many allergens (aeroallergens such as mite, pollen, cat, dog, fungal sp., and some food allergens) have protease activity. Proteolytically active allergens can directly (by proteolytic activity) and indirectly [by activation of pattern-recognition receptors (PRRs)] cause disruption of epithelial barrier and E-cadherin connections (Nawijn et al., 2011). Protease allergens lead to non-IgE-mediated airway reactions by triggering innate immune receptors such as PARs to activate epithelial cells, mast cells, and DCs. Thus, more mediators are released. It has been shown in previous studies that mite allergens induce proteolysis of ZO-1, occludin and other TJs proteins (Wan et al., 2001; Matsumura, 2012). In one of these studies performed in monolayers of MDCK (Madin-Darby canine kidney cell line), Calu-3 (human lung cancer cell line), and 16HBEC (human bronchial epithelial cell line), HDM (house dust mite) serine peptidases induced a progressive cleavage of TJs and increased epithelial permeability. Cleavage of TJs involved proteolysis of occludin and ZO-1 and was examined by immunoblotting and mass spectrometry. The increase in epithelial permeability was demonstrated by mannitol permeability (Wan et al., 2001). Similarly, proteases released by pollens have been shown to disrupt E-cadherin and TJ proteins such as occludin, claudin-1 (Vinhas et al., 2011; Hosoki et al., 2015). In addition proteases in mite, fungi and cockroach extracts activate the protease-activated receptor (PAR)1 and/or 2 which in turn leads to degradation of E-cadherin and AJ structure (Winter et al., 2006; Li et al., 2019). In some *in vitro* studies with pollen extracts, they have been shown to disrupt the junctional structure and epithelial function. For example, pollen extracts of Poa pratensis, Betula, Ambrosia induced loss of immunofluorescent labeling for the TJ proteins such as occludin, claudin-1, and ZO-1 on monolayers of MDCK and Calu-3 cells (Runswick et al., 2007). In a study by Vinhas et al. (2011) extracts of olive, cypress (Italian cypress), orchard grass (Dactylis glomerata) affected both epithelial junction function, and structure of Calu-3 cells. In contrast to these studies, there are no effect to epitelial barrier function in some reports. For example, Blume et al. (2013) showed no change in TEER (transepithelial electrical resistance) or expression of ZO-1 in human bronchial epithelial cells exposed to Phleum pretense. Aspergillus fumigatus proteinases also disrupt epithelial cell detachment by production of IL-6, IL-8, and monocyte chemoattractant protein 1 from airway epithelial cells (Chaudhary and Marr, 2011). And cockroach proteinases have been shown to cause disruption of the epithelial barrier by increasing IL-8 expression and PAR-2 activation (Page et al., 2003).

People are exposed to many cleaning products, laundry and dishwashing detergents in their daily lives. It has been shown in previous studies that detergents disrupt the integrity of the epithelium in the skin, respiratory tract and gastrointestinal tract and predispose to diseases associated with this condition (Medina-Ramon et al., 2005; Wang et al., 2019). Even in trace concentrations, anionic surfactants and detergents can disrupt the structure of TJs and damage the airway epithelial barrier function (Xian et al., 2016; Wang et al., 2019). The barrier function of the bronchial epithelial cells is damaged by inhalation of the residues of laundry detergents on clothing and floor surfaces. Wang et al. (2019) showed laundry detergents caused dose-related toxic effects on HBECs with irregular cell shape and leakage of lactate dehydrogenase after 24 h exposure. He also reported that laundry detergent exposure increased IL-33 levels on HBECs from healthy controls, asthmatic patients, and patients with COPD and increased TSLP on only HBECs from healthy controls (Chaudhary and Marr, 2011). This indicates that the T helper (TH) 2 immune response is induced with detergents.

Smoking is one of the most common and preventable health problems in Turkey and in the world. There are approximately 5,000 chemicals harmful to human health, mainly nicotine, in cigarette smoke (American Lung Association, 2019). Many studies have shown that exposure of cigarette smoke causes epithelial barrier dysfunction in the respiratory tract. As a result of this, cigarette smoke and nicotine exposure are associated with a decrease in TEER. This situation contributes to the pathogenesis of lung diseases such as asthma and chronic obstructive pulmonary disease (COPD). In a study with HBECs published in 2019, cigarette smoke extract (CSE) has been shown to decrease TEER and increase permeability in a concentration-dependent manner (Tatsuta et al., 2019). It was also reported that gene expression of E-cadherin, occludin, some of claudins (such as claudin-1, 3, 4, 7, 15) and ZO-1 were suppressed 12 h after CSE exposure. In addition, repeated exposure to cigarette smoke has been found to cause loss of epithelial barrier function and consequently epithelial mesenchymal transition (EMT) in human bronchial epithelial cells (Zou et al., 2013; Aghapour et al., 2018; Hou et al., 2019). Although e-cigarettes were initially marketed as a safer option than cigarettes, they have been shown to produce similar effects to airway epithelial barrier integrity and cause airway mucociliary dysfunction (Chung et al., 2019; Muthumalage et al., 2019).

Ozone is an air pollutant gas formed by the reaction of emissions from burned fossil fuels. Ozone is important in the homeostasis of airway epithelial barrier integrity. Acute ozone exposure causes epithelial barrier dysfunction, airway inflammation, peribronchial collagen deposition, and airway hyperresponsiveness (AHR). Recently, it has been shown in animal studies that ozone exposure increases the expression of e-cadherin and some other AJC proteins via IL-33 (Michaudel et al., 2018b; Sokolowska et al., 2019). Chronic ozone exposure also causes remodeling, fibrosis, and emphysema in the small airway (Michaudel et al., 2018a).

Particulate matter (PM) and diesel exhaust are also harmful to human respiratory health. In *in vitro* studies, it has been shown that PM may affect the airway epithelial barrier integrity by damaging structure of occludin, claudin-1, and ZO-1, and downregulating claudin-1 expression in airway epithelial cells (Caraballo et al., 2011; Liu et al., 2019). In a mouse model with BALB/c mice, exposure to PM particulate matter less than 2.5 μm has been resulted in decreased E-cadherin levels and increased IFN-γ, IL-2, IL-4, IL-6, and IL-10 in the bronchoalveolar lavage fluid (Chuang et al., 2015).

### Effect of the External Noxious Stimuli on Epithelial Barrier and E-Cadherin in Terms of Immune Response

The connection between epithelial cells and immune cells in tissues controls barrier function and homeostasis. In the event of disruption of epithelial integrity, adaptive and innate immune responses are activated through various cytokines and chemokines. Environmental factors such as protease allergens, detergents, air pollutants damage epithelial cells and induce the release of TSLP, IL-33 and IL-25 from the epithelium. These cytokines activate dendritic cells to stimulate TH2 cells and activate group 2 innate lymphoid cells (ILC2s). TSLP induces the conversion of naive T cells to the TH2 subtype in the presence of IL-4 in the medium. Type 2 immune response develops and type 2 cytokines (IL-4, IL-5, IL-9, IL-13) release. IL-5 stimulates eosinophils. Stimulates mucus production with IL-9. IL-4 and IL-13 stimulate IgE exchange in B cells, vascular endothelium for eosinophils, and TH2 cell migration. IL-33 and IL-25 also stimulate ILC2s and they induce eosinophils and a type 2 response. TH2 cells, ILC2s and their secreted cytokines IL-4 and IL-13 together open the epithelial TJ barrier. It has been reported to disrupt the bronchial epithelial barrier integrity, leading to increased permeability to fluorescein isothiocyanate-dextran and a decrease in TEER in human bronchial epithelial cell cultures (Wawrzyniak et al., 2017; Sugita et al., 2018). In addition, IL-33 has been shown to induce severe airway inflammation in wild-type and recombination-activating gene 2 (Rag2)^–/^^–^ (T and B cell–deficient) mice, but not any pulmonary inflammation in Rag2^–/–^Il2rg^–/–^ (ILC–deficient) mice. These results support that IL-33 induces airway inflammation due to ILC (Sugita et al., 2018).

On the other hand many of the signaling pathways activated by environmental stimuli cause transcriptional activation of nuclear factor (NF)-κB in the airway epithelium. NF-κB activity leads to loss of inhalation tolerance and induction of immunity (Cheng et al., 2007; Pantano et al., 2008). Loss of E-cadherin-mediated cell–cell contacts induces NF-κB signaling and increase proinflammatory activity in bronchial epithelium. Expression of TH2-mediated factors such as chemokine ligand (CCL)17 and TSLP increase (Heijink et al., 2007; Cowell et al., 2009). Thus, the balance between E-cadherin expression and NF-κB activity could constitute an epithelial molecular switch between a tolerogenic and a immunogenic phenotype.

## Role of E-Cadherin in Epithelial Barrier Disfunction, Epithelial Mesenchymal Transition and Mesenchymal Epithelial Transition

Many environmental factors such as allergens, air pollutants, especially cigarette exposure, damage the intercellular junction complexes and disrupt the airway epithelial and endothelial barrier function. Thus, bronchial and lung epithelial permeability increases. Increased epithelial permeability causes exposure to more allergens, smoking and air pollutants. In the pathogenesis of lung diseases such as asthma and COPD, this vicious circle can be blamed for the initiation of inflammation. In addition, a mechanism that affects the epithelial barrier in both asthma and COPD, called EMT, has been proposed.

EMT is defined as the transition of epithelial cells to mesenchymal phenotype and loss of epithelial function. With EMT, epithelial cell polarity is lost, the structure of junction complexes and cell-cell contacts are remodeled, and the actin cytoskeleton is reorganized. While prototypical markers of epithelial cells such as E-cadherin, occludins, and cytokeratins are lost, mesenchymal cell markers, such as N-cadherin, vimentin and α-smooth muscle actin (α-SMA), are obtained (Zou et al., 2013). In addition to its role in embryogenesis, EMT has been shown to be associated with wound healing, fibrosis and cancer processes in adult epithelial cells. Therefore, EMT plays a role in the pathogenesis of many lung diseases such as asthma, COPD, and lung fibrosis (Bartis et al., 2014).

Mesenchymal epithelial transition (MET) is a mechanism that plays a role in different stages of morphogenesis and organogenesis in the formation of the body plan of the organism. It occurs in intermittent alternation with EMT. MET is responsible for forming epithelium at different developmental stages in the embryonic period, and mesenchymal cells gradually acquire apicobasal polarity. Embryonic stem cells or induced pluripotent stem cells and somatic cells differentiate each other by sequential EMT-MET. MET is a critical step for pluripotency (Shu and Pei, 2014). MET can also be harmful to tissue in the onset of asthma and airway inflammation. Because the epithelial cell will transform into myofibroblast and try to compensate the epithelial barrier dysfunction. However, once the stress causing epithelial barrier dysfunction is removed, myofibroblastic transformation by MET will revert to the old normal airway epithelium for normal epithelial regeneration (Kim et al., 2017).

**FIGURE 4:**
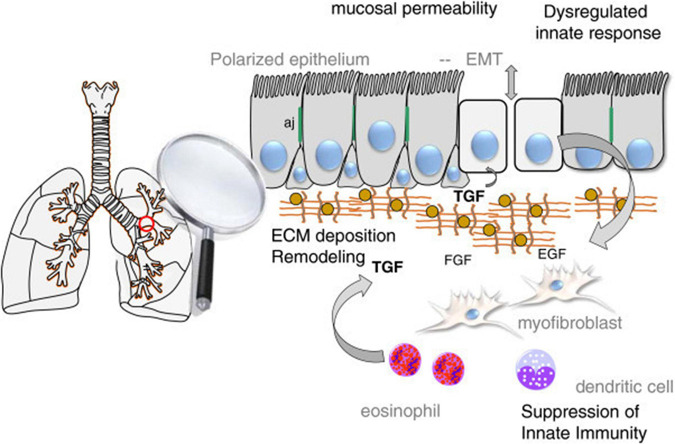
Schematic view of EMT in the airway.

EMT plays a role in the pathogenesis of many lung diseases such as asthma, COPD and lung fibrosis. It has also been shown to trigger EMT in relation to hypertrophy, metaplasia, gene mutation, and modification of lung epithelial cells, which are considered to be a critical mechanism in the pathogenesis of smoking COPD patients (Milara et al., 2013). EMT is also recommended in asthma as a mechanism affecting the epithelial barrier. In the respiratory tract of healthy individuals, epithelial cells send signals to mesenchymal cells in the subepithelial area and an epithelial-mesenchymal unit (EMU) is formed. Growth factors secreted from epithelial cells ensure the integrity of EMU. Airway epithelial integrity is impaired in asthma. The secretion of growth factors increases in response to epithelial damage to regenerate the epithelial surface. As a result of epithelial disruption, epithelial basement membrane integrity is lost and it initiates epithelial cell activation by growth factors [such as epidermal growth factor (EGF), fibroblast growth factor (FGF)] and cytokines (such as IL-6, IL-1β, especially TGF-β). Especially TGF-β has a critical inducer role to transform epithelial cells into mesenchimal cells (Figure 4; Ijaz et al., 2014). Although EMT is a protective mechanism for tissue repair, exaggerated and prolonged EMT process can lead to fibrosis and tissue damage (Kim et al., 2006).

There is also some other evidence that EMT occurs in asthmatic epithelial cells and contributes to airway remodeling. HDM extracts have been reported to induce EMT properties in human bronchial epithelial cell lines, particularly in accordance with TGF-β (Heijink et al., 2010b). Johnson et al. (2011) showed that chronic HDM exposure caused that loss of E-cadherin and occludin and was detected EMT findings in bronchial epithelial cells of mice. In another study, it was observed that low E-cadherin levels in sputum were associated with asthma severity. This is an indication of the loss of E-cadherin in asthma. Consequently, previous studies suggest that EMT and epithelial differentiation are related with epithelial barrier function, but more studies are needed to understand this interaction.

## Regulation of Respiratory Epithelial Integrity During Airway Diseases and Role of E-Cadherin

### Asthma

Asthma is one of the most common chronic inflammatory diseases in both children and adults. Its frequency is increasing day by day. Asthma is a heterogeneous disease with different clinical course, occurrence and treatment response in each subtype. Airway inflammation, AHR, airway remodeling, and mucus hypersecretion, which are caused by a trigger, mainly play a role in the pathogenesis of asthma (Taylor et al., 2008). Recently, due to the complexity and variety of symptoms, different subtypes or endotypes of asthma have been identified. Most asthmatic patients have allergic inflammation of the airways, and this process defines the “TH2 high” subtype. In sensitized individuals, TH2 cells initiate allergic inflammatory process by secreting some cytokines such as interleukins (IL) –4, –5, –9 and –13, and granulocyte-macrophage colony stimulating factor (GM-CSF) (Frey et al., 2020). Thus, allergen-specific immunoglobulin E response occurs, eosinophils activate, TH2 cells develop and mature. Goblet cell differentiation and submucosal gland activity are also increased through these mediators (Papi et al., 2018). In brief, allergic sensitization to aeroallergens and the resulting hyperreaction of the immune system play a central role in the pathogenesis of asthma.

In recent years, experimental studies have come to the fore in studies on asthma. Because of the allergic inflammatory response in asthma, increased TH2 cytokines. Because of the allergic inflammatory response in asthma, increased TH2 cytokines such as IL-4 and –13 inhibit expression of E-cadherin, occludin and ZO-1 (Soyka et al., 2012; Saatian et al., 2013). Mast cell-derived mediators are also thought to have an impact on airway epithelial function. Zabner et al. (2003) showed that histamine disrupts the structure of the apical junction complex and increases epithelial permeability and TEER in ECA304 (human urinary bladder carcinoma cell line). TSLP is an important cytokine in the epithelial response of asthma. In a study with asthmatic BALB/c mice and 16HBE cells, HDM increased the expression of TSLP, and E-cadherin dysfunction by PI3K/Akt signaling pathway (Hu et al., 2017). In this study firstly 16HBE cells were stimulated with HDM for the indicated times (0, 2, 4, 8, and 12 h) and for the indicated concentrations (0, 200, 400, 800, and 1,600 U/ml). TSLP expression was examined. HDM (400 U/ml) caused a significant increase in the protein expression levels of TSLP (for 8 h). 16HBECs were treated with PI3K inhibitor and partially inhibited these changes. Then it was shown that exposure of 16HBE cells to HDM (400 U/ml) and TSLP (10 ng/ml) resulted in abnormal distribution and cleavage of E-cadherin. In HDM-induced asthmatic mice model, it was reported that airway hyperreactivity and airway inflammation were alleviated by TSLP neutralization. In a study by Saatian et al. (2013) the effects of TH2 cytokines on HBEC epithelial barrier function were investigated. TEER and FITC-conjugated dextran (3 kDa) were applied for epithelial permeability. TEER decreased and 3 kDa dextran permeability increased and AJ/TJ proteins such as E-cadherin, β-catenin, ZO-1, occluding, and claudin-4 decreased in cells treated with IL-4. Similar results were obtained with IL-13, but TSLP, IL-25, and IL-33 did not significantly affect barrier function.

Airway epithelial cells are the first line of defense to encounter environmental stressors such as allergens, bacterial and viral pathogens, and air pollutants in the airway. Epithelial cells both act as a barrier with cell-to-cell connections and provide neutralization of these factors through mucociliary clearance mechanism. E-cadherin plays an important role in the binding of actin filaments of epithelial cells to each other in this process. Disruption of epithelial barrier function has been strongly demonstrated in many studies conducted with asthma patients. It has been shown in bronchial biopsies of asthmatic individuals that the expression of an array of proteins required for the formation of TJs and AJs is significantly reduced.

Especially respiratory tract viruses cause connection dysfunction in epithelial cells by different mechanisms. Respiratory viruses such as respiratory syncytial virus (RSV), rhinoviruses, influenza, and parainfluenza bind to their entry receptors, which are protein or sugar structures, for endocytosis (Frey et al., 2020). After the virus is internalized, the viral replication process begins. As a result of the cellular immune response that slows virus replication, type I interferons (IFN) are released. Infected epithelial cells are killed by cytotoxic CD8 + T cells or by the virus’s own cytopathic action. Previous studies have shown that rhinovirus and RSV disrupt the airway epithelial barrier structure by down-regulating epithelial junction proteins such as ZO-1 and occludin (Singh et al., 2007; Faris et al., 2016; Looi et al., 2016). Similarly, it has been reported that RSV disrupts AJC structures by increasing the activity of protein kinase D (PKD) (Rezaee et al., 2011).

Allergens, viruses, air pollutants and the inflammatory response to their exposure are external factors that disrupt the epithelial barrier integrity in asthma. Furthermore, some evidence suggests that epithelial cells naturally predispose to increased permeability in asthma patients. Airway epithelial cells isolated *in vitro* from asthmatic patients exhibit a low TEER compared to cells from healthy donors as an indication of disruption of epithelial barrier integrity (Post et al., 2013; Faris et al., 2016).

The cell-cell adhesion molecule E-cadherin plays an essential role in the formation of epithelial junction. Function and gene expression of E-cadherin are as important as the presence of E-cadherin for the airway epithelial barrier integrity. There is evidence showing a relationship between single nucleotide gene polymorphism and airway remodeling, inflammation and lung function in the pathogenesis of asthma. A single nucleotide polymorphism at position 160 in the CDH1 gene promoter has been identified as C/A. In a study by Wang et al. (2013), CDH1 AA/CA genotype has been shown to result in decreased E-Cadherin gene transcription. In another study, of 17 patients with SNPs, seven were associated with airway remodeling, three with CD8^+^ T cell counts, two with eosinophil counts, and seven with decreased FEV1 (Lerodiakonou et al., 2011). Based on these observations, it is not clear whether there is an encoded predisposition in epithelial stem cells or an epigenetic modification in the epithelial cells of asthma patients.

### Chronic Obstructive Pulmonary Disease

COPD is a disease characterized by permanent airflow restriction caused by chronic inflammation of the airways. The main findings in tissue are epithelial remodeling and subepithelial fibrosis of small airways. Smoking is the major predisposing factor for COPD. Not just smoking other environmental noxious agents and genetic predispositions also contribute COPD development. Smoking causes oxidative stress in the airway epithelium (van der Toorn et al., 2009). This can lead to migration of immune cells to the scene and initiation of the inflammatory process, with subsequent destruction of the matrix and epithelial cell damage (Houghton, 2013). Thus, squamous metaplasia develop and hypersecretion of mucus and loss of ciliary beating add to the event. In addition, smoking, which plays an important role in the pathogenesis of COPD, disrupts the junctions between epithelial cells (Heijink et al., 2012; Schamberger et al., 2014).

The barrier function, provided by AJC is disrupted in COPD. E-cadherin and zonula occludens proteins such as ZO-1 and occludin are decreased both *in situ* in the native airway epithelium from COPD patients and *in vitro* in derived air/liquid interface (ALI)-cultured epithelium. In a research in 16HBECs and primary bronchial epithelial cells (PBECs) from COPD patients, non-smokers, and healthy smokers, it was shown CSE rapidly and tranciently impairs barrier function of 16HBEs. CSE disrupts epitelial cell–cell contacts. CSE induced a similar, but stronger and more sustained, defect in PBECs of COPD patients (Heijink et al., 2012). In a study done in the last year, primary cultures of ALI from non-smoker controls, smoker controls, and COPD patients were assessed in very 10 weeks. Epithelial defects, barrier dysfunction and impaired polarity persisted up to 10 weeks in smokers and COPD. In addition, TNF-α, IL-6, and IL-1β treatment induced COPD-like changes and was able to reactivate epithelial-to-mesenchymal transition in COPD cells (Carlier et al., 2021).

**FIGURE 5:**
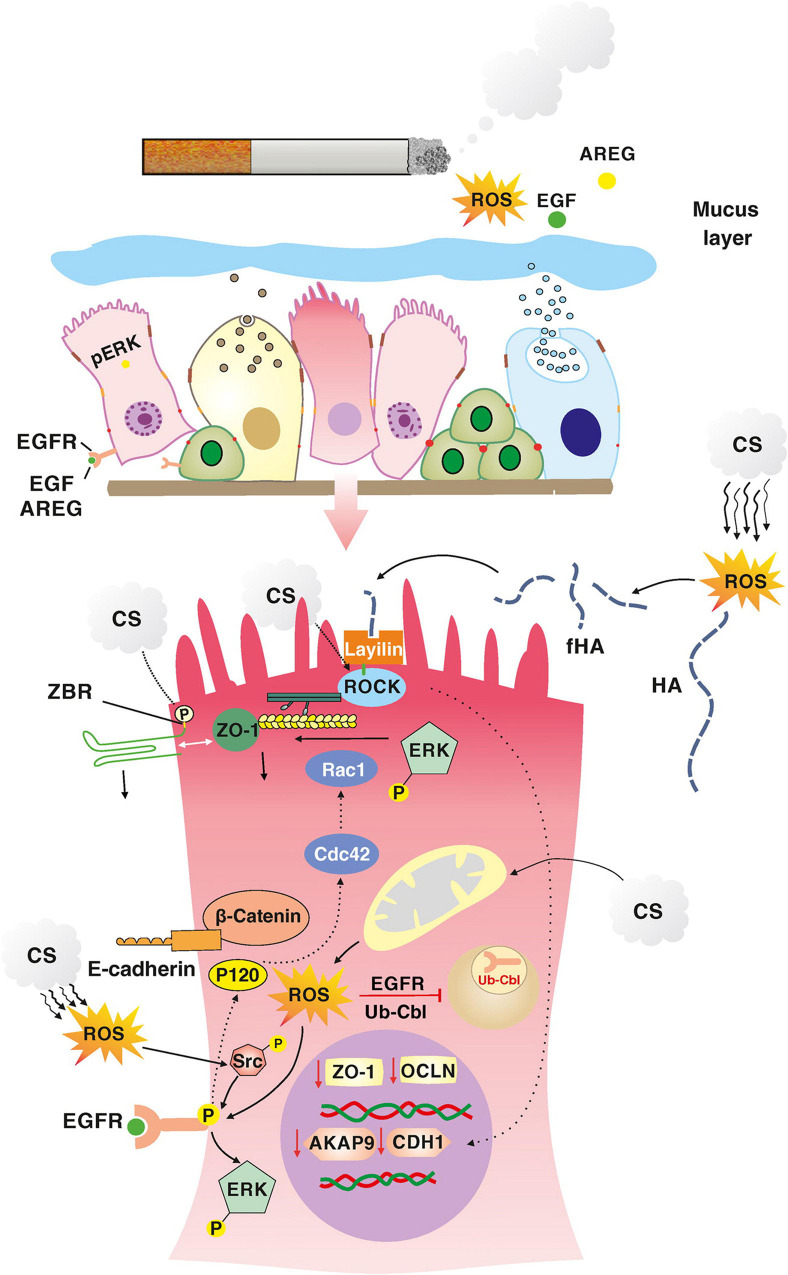
The molecular effect of external noxious on epithelial cell.

Smoking is an major cause of developing COPD. In a study by Shaykhiev et al. (2011). Healthy and COPD patients were grouped as smokers and non-smokers. Small airway epithelial cells of these patients were collected by brushing with fiberoptic bronchoscopy. The transcriptional program that regulates airway epithelial AJC integrity was analyzed. Transcriptome analysis revealed global down-regulation of physiological AJC gene expression in the airway epithelium of healthy smokers compared to non-smokers. The overall expression of AJC-related genes was further decreased in COPD smokers. Exposure of airway epithelial cells to CSE *in vitro* resulted in down-regulation of several AJC genes and decreased TEER. Smoking down-regulates A-kinase-binding protein (AKAP)-9 (Oldenburger et al., 2014), thereby decreasing E-cadherin and causing epithelial barrier dysfunction in COPD. AKAP-9 regulates protein kinase (PK) A, which is responsible for the localization of E-cadherin to the basolateral membrane. When epithelial cells were exposed to smoking, it was induced EGFR and downstream extracellular signaling the kinase (ERK) activation (Petecchia et al., 2009; Heijink et al., 2010c; Zuo et al., 2017). On the other hand, ROS is in cigarette smoke can induce Rho kinase (ROCK) phosphorylation via its surface receptor layilin. ROCK activation can damage AJCs and decrease E-cadherin expression (Forteza et al., 2012; Aghapour et al., 2018; Figure 5).

E-cadherin downregulation also initiates the EMT process in COPD. In this process, both with the effect of nicotine itself and oxidative stress caused by cigarette smoke lead to TGF-β activation and wingless/integrase-1 (WNT) signaling. E-cadherin is down-regulated, mesenchymal cell markers increase. Thus the epithelial phenotype changes and EMT occurs (Milara et al., 2013; Zou et al., 2013; Gohy et al., 2015).

It is known that both innate and adaptive immunity play a role in regulating airway epithelial barrier function. Dysfunction of airway epithelial junctions is a defining feature in chronic inflammatory airway diseases such as COPD. Especially TH 2 and 17 cells and cytokines released from these cells are critical factors for epithelial integrity (Saatian et al., 2013; Ramezanpour et al., 2016). In this respect, *in vitro* studies on airway epithelial cells have shown that exposure to TH2 cytokines such as IL-4 and IL-13 activates Janus-associated kinase (JAK) and increases epithelial permeability (Saatian et al., 2013). Th17 cells and IL-17 levels released by these cells were found to be higher in the airway of patients with COPD compared to healthy controls (Doe et al., 2010; Vargas-Rojas et al., 2011; Zhang et al., 2013). In addition, proinflammatory cytokines such as TNF-α, IFN-γ, and IL-1β have been found to contribute to barrier dysfunction in COPD.

### İdiopathic Pulmonary Fibrosis

Idiopathic pulmonary fibrosis is a chronic lung disease characterized by irregular wound healing in the lung tissue and consequently abnormal fibroblast accumulation. It is a histopathologically heterogeneous disease with areas of paraseptal thickening and subpleural fibrosis interspersed between normal parenchymal areas (The Idiopathic Pulmonary Fibrosis Clinical Research Network, 2012). IPF is characterized by the onset of alveolar epithelial damage, the increase in inflammatory mediators, and the consequent increase in pro-fibrotic cytokine expression, extracellular matrix deposition, and the development of fibrotic areas in the lung parenchyma (Wilson and Wynn, 2009). In IPF, the phenotype of alveolar epithelial cells alternate. Although the main reason that initiated the fibrotic process is still unknown, apoptosis and/or senescence of epithelial cells seems to be a rcause of the events. Continuous epithelial barier disruption and concomitant apoptosis of epithelial cells contribute to the fibrosis (Jin and Dong, 2011).

Under normal conditions, when the epithelium is damaged, the fibroblastic cascade is activated and the number of ATI cells is reduced. ATII cells can differentiate into ATI as needed and repair epithelium when epithelial integrity is disrupted. Thus, ATII cells undergo hyperplasia. In IPF lung, hyperplasia of ATII cells is severely impaired. Due to the absence of hyperplasia in ATII cells, the basement membrane structure is disrupted, the alveoli collapse and the integrity of the alveolar epithelial barrier is impaired.

There are many genetic and environmental factors that trigger apoptosis in epithelial cells. Genetic factors such as mutations in the telomerase enzyme and surfactant protein C (SP-C) may be a cause of cell senescence (Chilosi et al., 2012; Noble et al., 2012). Environmental factors such as chronic exposure to toxic substances, especially smoking (Tsuji et al., 2004; Psathakis et al., 2006), infections especially with viral agents (Tsukamoto et al., 2000) and gastroesophageal reflux (Lee et al., 2012) are also some of the triggers currently being investigated (Camelo et al., 2014).

**FIGURE 6:**
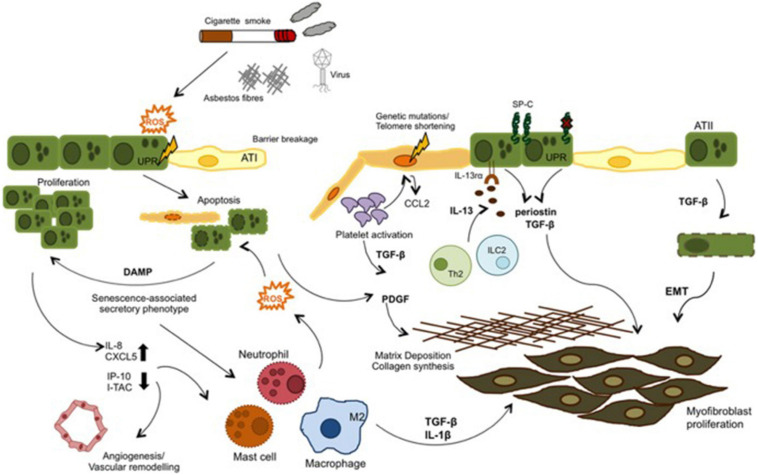
Mechanisms of external noxious on epithelia mesenchymal transition.

External irritants such as cigarette smoke, asbestos, etc. stimulate oxidative stress and causes ROS production in IPF. ROS can both induce apoptosis by increasing endoplasmic reticulum stress in epithelial cells and initiate the inflammatory process by being released from damaged epithelium together with chemokines and cytokines such as TGF-β. Leukocytes are called into the tissue and the coagulation pathway is activated. In IPF these mechanisms are dysregulated. Inflammatory cells both stimulate repair and contribute to unresolved damage. TGF-β is also induce EMT. As a result fibroblast proliferation and ECM deposition develop (Figure 6; Camelo et al., 2014).

### Acute Respiratory Distress Syndrome

Acute respiratory distress syndrome (ARDS) is a disease in which acute respiratory failure associated with diffuse pulmonary infiltrates. In the pathogenesis of ARDS, the alveolar capillary barrier is disrupted, followed by leakage of proteinaceous tissue fluid into the alveolar area and extensive fibrosis (Matute-Bello et al., 2008; Matthay et al., 2012).

Disruption of the alveolar epithelial integrity, abnormal activation of alveolar epithelial cells, and abnormal repair of alveolar epithelium play a crucial role in the pathogenesis of IPF and ARDS. Some of the mechanisms that may be responsible for the abnormal activation of AECs shown by previous studies are as follows: disruption of the molecules essential for epithelial integrity, recapitulation of the developmental pathway, and the acceleration of senescence features (Camelo et al., 2014). In ARDS and pulmonary fibrosis, one of the main mechanisms of maintenance and reconstitution of AECs integrity is also epithelial Pten/PI3K/Akt pathway. In previous studies Pten-deficient airway epithelial cells it has been shown that expression of ZO-1, claudin 4, and laminin is decreased (Yanagi et al., 2015).

E-cadherin-targeted new therapies are being investigated more in the field of cancer disease. By increasing E-cadherin, inhibition of EMT and anticancer activity is provided. As far as can be seen, there are no data yet on the use of E-cadherin in the treatment and management of pulmonary diseases with epithelial barrier dysfunction. Conducting new experimental studies on E-cadherin-targeted therapies in lung diseases due to respiratory barrier dysfunction such as COPD, idiopathic pulmonary fibrosis and ARDS will support this issue.

## Conclusion

In the light of these data, E-cadherin is a very important regulatory protein in the establishment, and maintenance of epithelial cell-cell contacts and in the formation of airway epithelial barier. E-cadherin dysfunction and consequently epithelial barrier dysfunction play a role in the pathogenesis of many respiratory diseases such as asthma, COPD, idiopathic pulmonary fibrosis, and ARDS. Prospective experimental models regarding the role and regulation of e cadherin in epithelial barriers may contribute to this issue. Better understanding of E-cadherin in respiratory barrier dysfunction-induced lung diseases may shed light on the identification of specific biological targets to open new therapeutic perspectives.

## Author Contributions

HY was the main planner, coordinator, and correspondent of this article. MO was the literature review task and was responsible for the writing of the first three main chapters. OY was the literature review task and was responsible for the writing of the last three main chapters. All authors contributed to the article and approved the submitted version.

## Conflict of Interest

The authors declare that the research was conducted in the absence of any commercial or financial relationships that could be construed as a potential conflict of interest.

## Publisher’s Note

All claims expressed in this article are solely those of the authors and do not necessarily represent those of their affiliated organizations, or those of the publisher, the editors and the reviewers. Any product that may be evaluated in this article, or claim that may be made by its manufacturer, is not guaranteed or endorsed by the publisher.
